# Quantification of Medical Waste and Incineration‐Related CO_2_ Emissions Associated With Gastrointestinal Endoscopy at Two Facilities in Japan: A Preliminary Observational Study

**DOI:** 10.1002/deo2.70455

**Published:** 2026-10-22

**Authors:** Ryosuke Kawanishi, Atsushi Imagawa, Naoya Tada, Noriko Nishiyama, Haruo Oba, Hideki Kobara

**Affiliations:** ^1^ Department of Gastroenterology and Neurology, Faculty of Medicine Kagawa University Kagawa Japan; ^2^ Department of Gastroenterology Imagawa Medical Clinic Kagawa Japan; ^3^ Faculty of Engineering and Design Kagawa University Kagawa Japan

**Keywords:** carbon dioxide emissions, gastrointestinal endoscopy, green endoscopy, medical waste, sustainable development goals

## Abstract

**Objectives:**

Sustainable development oals in gastrointestinal endoscopy (GIE), referred to as “green endoscopy,” have been a research focus in Western countries. However, the environmental burden of GIE in Japan is poorly understood. We quantify medical waste and estimate GIE‐derived incineration‐related CO_2_ emissions in Japan.

**Methods:**

In a dual‐center observational study, we report waste from esophagogastroduodenoscopy (EGD), colonoscopy (CS), endoscopic retrograde cholangiopancreatography (ERCP), and endoscopic submucosal dissection (ESD) from June 2024 to January 2025. We classify waste as infectious/non‐infectious, combustible/non‐combustible, liquid, or sharps. We record the per‐procedure waste and the procedure time, estimate CO_2_ emissions from the combustion of the measured waste mass using combustion equations for cellulose and polyethylene, and calculate per‐minute emissions and annual totals. Sensitivity analyses are performed for changes in the anticipated waste composition.

**Results:**

Estimated per‐procedure CO_2_ emissions are 949, 1178, 5834, and 4780 g for EGD, CS, ESD, and ERCP, respectively. Aggregate per‐minute emissions are 168, 165, 89, and 75 g/min for ERCP, EGD, CS, and ESD, respectively. Annual institutional emissions total approximately 5800 kg. Estimated emissions are 64%–69% and 123%–134% of the base composition scenario values in the lower‐ and higher‐emission composition scenarios, respectively.

**Conclusions:**

ESD had the highest per‐procedure emissions, and ERCP and EGD had the highest per‐minute emissions. These results provide preliminary baseline data on incineration‐derived CO_2_ emissions as a foundation for promoting “green endoscopy.”

**Abbreviations**
CS: colonoscopy
EGD: esophagogastroduodenoscopy
ERCP: endoscopic retrograde cholangiopancreatography
ESD: endoscopic submucosal dissection
GIE: gastrointestinal endoscopy.

## 1 Introduction

Climate change increasingly threatens global health. Paradoxically, healthcare also contributes substantially to greenhouse gas emissions through energy consumption, transportation, and the generation of medical waste [1]. Japan, with a rapidly aging population, has had difficulty maintaining a sustainable healthcare system, and its government has set targets to reduce greenhouse gas emissions by 46% from 2013 levels by 2030 and to achieve carbon neutrality by 2050 [2].

Gastrointestinal endoscopy (GIE), one of the most frequently performed examinations at healthcare facilities, represents the third largest source of hospital‐activity‐related medical waste [3]; disposable devices are widely used in GIE to maintain sterility and safety [4]. However, the high daily volume of procedures (e.g., esophagogastroduodenoscopy [EGD] and colonoscopy [CS]) means that the cumulative environmental burden of GIE is considerable. Therefore, “green endoscopy” was introduced by the European Society of Gastrointestinal Endoscopy and other organizations to promote environmentally sustainable endoscopic practices through initiatives such as carbon footprint assessment, waste reduction, and workflow optimization [5].

Green endoscopy is broadly supported by endoscopy staff in the Asia–Pacific region but is rarely implemented in practice [6]. Despite the increased frequency of GIE procedures, few studies have quantitatively evaluated the characteristics and extent of GIE‐generated medical waste [7], particularly in East Asian countries, including Japan. Accordingly, to identify methods to improve sustainability in clinical practice, we quantify the medical waste generated from routine upper and lower GIE procedures in Japan and subsequently estimate the associated incineration‐related CO_2_ emissions to provide preliminary baseline data to support more environmentally sustainable endoscopy practices.

## 2 Methods

### 2.1 Survey Methodology

Surveys were undertaken between June 2024 and January 2025 at a secondary medical institution (Kagawa Rosai Hospital) and a university hospital (Kagawa University Hospital) in Japan. A total of 137 GIEs were audited: 131 at Kagawa Rosai Hospital (79 EGDs, 38 CSs, 10 endoscopic retrograde cholangiopancreatographies [ERCPs], and four endoscopic submucosal dissections [ESDs]) and six ESDs at Kagawa University Hospital, making ESDs the only type of procedure measured at both institutions.

All the procedures were performed by a single operator (Ryosuke Kawanishi), and the disposable devices used were essentially identical; the classification and segregation of the waste, and the subsequent disposal and incineration pathways, were identical at the two institutions. Data from both institutions were, therefore, combined.

For each audited procedure, the duration from the endoscope's insertion to its complete withdrawal was recorded using a digital timer, and the medical waste was weighed on a calibrated electronic scale with a readability of 2 g, at the level of aggregation described in the next paragraph. Waste was categorized as infectious (combustible and non‐combustible), non‐infectious (combustible and non‐combustible), liquid (such as suctioned fluids), and sharps or other materials designated for a landfill. Each category was quantified separately; liquid waste was recorded by volume and converted to mass, assuming a density of 1 g/mL. Infectious waste comprised waste contaminated with patients’ body fluids; non‐infectious waste included packaging materials for medical devices. All the disposable items used or generated during each procedure were collected and weighed (e.g., biopsy forceps, sheaths, snares, knives, syringes, gauze, drapes, and disposal bags). Personal protective equipment used outside the procedure room was not included.

The duration of each procedure was recorded in all four groups, using the same definition throughout. Waste was collected only from the audited procedures—those performed by the first author and for which the procedure duration was measured—and not from other procedures performed in the same room. For ESDs and ERCPs, the waste was weighed separately for each case; for EGDs and CSs, it was pooled and weighed as a single daily total across 16 and 15 measurement days, respectively, so that attribution to individual cases was impossible. Per‐procedure and time‐adjusted values for EGDs and CSs are, therefore, derived from these daily totals.

This study was approved by the Clinical Ethics Committee of Kagawa University Hospital (Registration No. 2025–074) and was performed in accordance with the Declaration of Helsinki. Kagawa Rosai Hospital determined that formal ethics review was not required at that institution (see Ethics Statement).

### 2.2 Evaluation Parameters

The total amount of waste was calculated based solely on weight (g), including landfill waste. CO_2_ emissions were estimated from the per‐endoscopy waste (excluding non‐incinerated liquid and landfill wastes) by modeling waste incineration. This analysis does not represent the complete carbon footprint of each procedure. At both study institutions, both infectious and non‐infectious medical wastes (combustible or otherwise) were incinerated. Accordingly, combustible and non‐combustible wastes were approximated as cellulose (C_6_H_10_O_5_)*n* and polyethylene (CH_2_)*n*, respectively.

CO_2_ emissions were estimated using the following combustion equations [8]:

(C_6_H_10_O_5_)*n* + 6*n*O_2_ → 6*n*CO_2_ + 5*n*H_2_O

(CH_2_)*n* + 1.5*n*O_2_ → *n*CO_2_ + *n*H_2_O

The waste and estimated CO_2_ emissions per minute of endoscopy were calculated by dividing the total values by the procedural duration (in minutes), and the annual CO_2_ emissions by multiplying the aggregate per‐procedure estimates by the annual procedure volumes. Because almost all the measurements were obtained at Kagawa Rosai Hospital, the primary annual estimate was calculated using the annual procedure volume of that institution (2314 EGDs, 1753 CSs, 295 ERCPs, and 21 ESDs).

### 2.3 Sensitivity Analysis

To evaluate the robustness of the estimated CO_2_ emissions relative to the chemical approximations used, a scenario‐based sensitivity analysis was performed. The three scenarios were a base composition scenario, using the chemical approximations (where combustible and non‐combustible wastes were approximated as cellulose and polyethylene, respectively); a lower‐emission composition scenario, where all the incineration‐eligible waste was assumed to behave as cellulose; and a higher‐emission composition scenario, where all such waste was assumed to behave as polyethylene. The same combustion equations were applied. Liquid and landfill‐designated wastes were excluded from each scenario.

Importantly, this analysis only addresses the uncertainty in the assumed chemical composition of the incinerated waste. Other sources of uncertainty—including alternative disposal pathways, transport distances, moisture content, combustion efficiency, and energy recovery—were not varied, and the scenarios, therefore, do not represent the overall lower and upper bounds of the uncertainty in the estimated emissions.

### 2.4 Statistical Analysis

All the statistical analyses were performed using JMP Pro v17.0 (SAS Institute Inc., Cary, NC, USA). The amounts of waste and per‐procedure and per‐minute CO_2_ emissions were calculated in MS Excel. Throughout the text and tables, *n* denotes the total number of procedures and *m* the number of measurement days.

For each procedure type, the principal per‐procedure and time‐adjusted values are aggregate estimates, calculated as (the total waste mass or total estimated CO_2_ emissions) divided by (the total number of procedures) or (the total procedure duration in minutes) across all the measurements. These are aggregate ratios and are not means of case‐ or day‐level values.

To describe variability, case‐ and day‐level values were also derived. For ESDs and ERCPs, the waste mass, estimated CO_2_ emissions, and per‐minute rates were calculated for each case. For EGDs and CSs, the following values were calculated for each measurement day: the daily total waste divided by the number of procedures performed that day, and the daily total waste or estimated CO_2_ emissions divided by the total procedure duration on that day. The distributions of these case‐ and day‐level values, and the number of procedures performed per measurement day, are summarized as mean ± standard deviation (SD) with the observed range. Because case‐level waste data were not available for EGDs and CSs, the distributions reported for both procedures describe the variability across measurement days and do not imply case‐level precision.

No inferential tests or group comparisons were performed because our objective was to provide preliminary descriptive baseline data for environmental impact assessments.

## 3 Results

### 3.1 Amounts of Waste and Per‐Examination and Per‐Procedure CO_2_ Emissions

On average, EGDs, CSs, ESDs, and ERCPs generated 626, 997, 3406, and 2707 g of waste, respectively. Estimated incineration‐related per‐procedure CO_2_ emissions (excluding landfill‐designated and liquid wastes) were the highest for ESDs (5834 g), followed by ERCPs, CSs, and EGDs (4780, 1178, and 949 g, respectively). Among the waste categories, non‐combustible infectious waste accounted for the highest mass in both ESDs and ERCPs. The waste designated for landfill disposal (e.g., electrosurgical units and puncture needles) was the highest in ESDs (averaging 308 g per procedure).

A mean of 4.9 ± 1.5 EGDs (range 2–8) and 2.5 ± 1.0 CSs (range 1–5) were performed on each measurement day. Across the 16 EGD measurement days, the amount of day‐level per‐procedure waste was 644 ± 122 g (range 450–847), and the corresponding estimated CO_2_ emissions were 981 ± 181 g (range 713–1368). Across the 15 CS measurement days, the corresponding values were 1047 ± 297 g (range 766–2018) and 1200 ± 232 g (range 954–1718), respectively. The wide range for CSs reflects a single measurement day on which only one procedure was performed, and 1550 of the 2018 g of waste recorded was liquid.

The quantities of each waste category, including the case‐level distributions for ESDs and ERCPs, are detailed in Table 1. Representative waste from a single ESD is illustrated in Figure 1.

**TABLE 1:** Waste (g) and estimated CO_2_ emissions (g) generated per gastrointestinal endoscopy at two medical institutions. For esophagogastroduodenoscopies (EGDs) and colonoscopies (CSs), the per‐procedure value represents an aggregate estimate calculated as (the total waste across all the measurement days)/(the total number of procedures across all the measurement days). For endoscopic submucosal dissections (ESDs) and endoscopic retrograde cholangiopancreatographies (ERCPs), values are presented as the mean ± standard deviation per case (*n* = 10 for each). The lower block shows the distribution of these values across *m* measurement days (EGDs and CSs) or *n* cases (ESDs and ERCPs). The values were calculated from unrounded data and may not sum exactly to the totals.

Waste category	EGD (*n* = 79, *m* = 16)	CS (*n* = 38, *m* = 15)	ESD (*n* = 10)	ERCP (*n* = 10)
***Waste and estimated CO_2_ emissions per procedure (g): aggregate estimate (EGD, CS) / case‐level mean ± SD (ESD, ERCP)***				
Combustible infectious waste	183	143	458 ± 161	595 ± 159
Combustible non‐infectious waste	28	36	538 ± 178	406 ± 143
Non‐combustible infectious waste	173	253	1208 ± 285	833 ± 350
Non‐combustible non‐infectious waste	19	29	131 ± 64	169 ± 46
Liquid waste^a^	209	492	762 ± 784	630 ± 267
Landfill‐designated waste^a^	13	44	308 ± 206	74 ± 78
Total waste	626	997	3406 ± 899	2707 ± 506
Estimated incineration‐related CO_2_ emissions^b^	949	1178	5834 ± 1057	4780 ± 1064
***Variability across measurement days (EGD, CS) or across cases (ESD, ERCP)***				
Procedures per measurement day, mean ± SD (range)	4.9 ± 1.5 (2–8)	2.5 ± 1.0 (1–5)	−	−
Total waste per procedure, mean ± SD (range) (g)	644 ± 122 (450–847)	1047 ± 297 (766–2018)	3406 ± 899 (2270–5440)	2707 ± 506 (2170–3854)
Estimated CO_2_ per procedure, mean ± SD (range) (g)	981 ± 181 (713–1368)	1200 ± 232 (954–1718)	5834 ± 1057 (4222–7610)	4780 ± 1064 (3622–6582)

^a^ Liquid and landfill‐designated wastes are excluded from the estimation of CO_2_ emissions.

^b^ Estimated CO_2_ emissions are incineration‐related only, derived from the chemical approximation of combustible waste as cellulose (C_6_H_10_O_5_)*n* and non‐combustible waste as polyethylene (CH_2_)*n*, and do not represent the full carbon footprint of each procedure (see Methods and Discussion).

**FIGURE 1:**
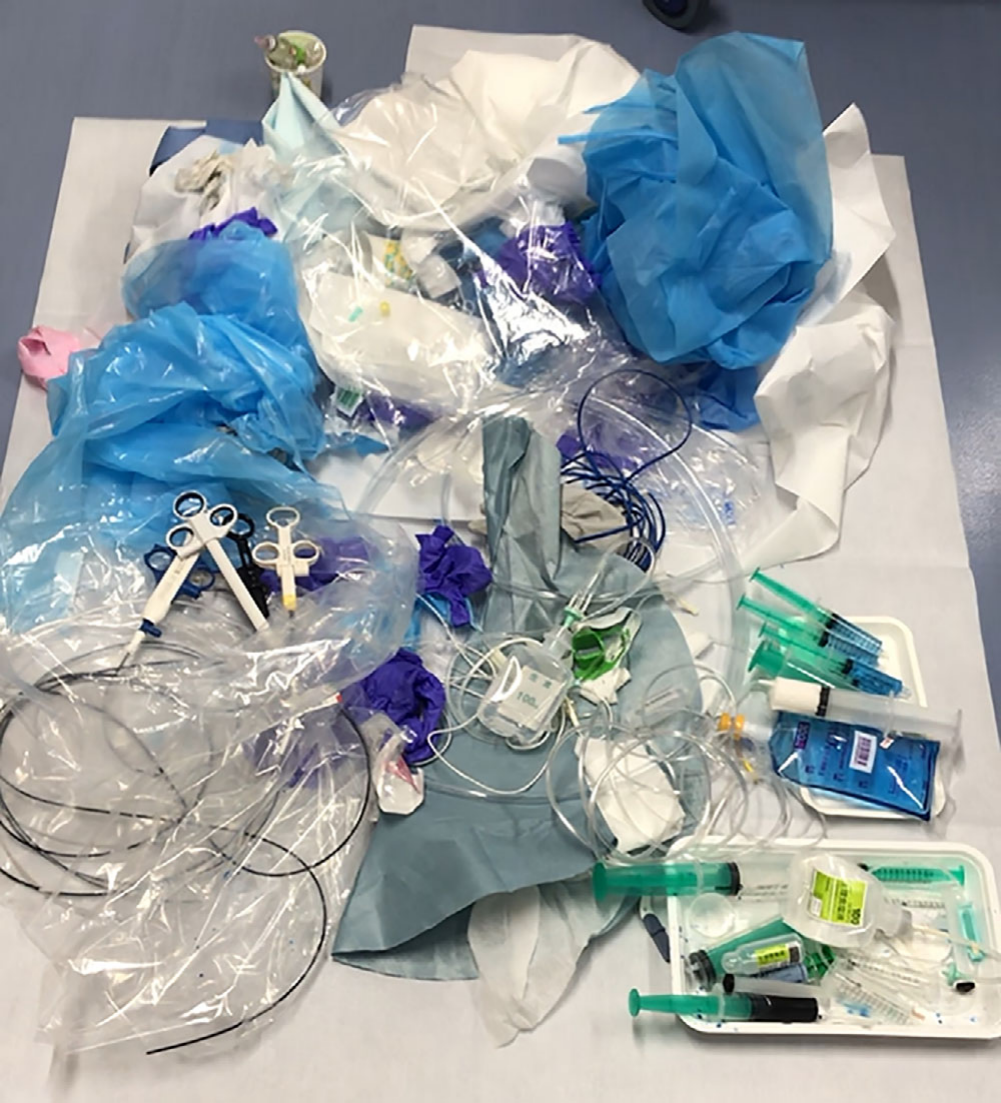
Medical waste generated during a representative endoscopic submucosal dissection (ESD).

### 3.2 Waste and CO_2_ Emissions Generated Per Unit of Procedure Time

When adjusted for procedure duration, the aggregate estimated CO_2_ emission rate was the highest for ERCPs (168 g/min), followed by EGDs, CSs, and ESDs (165, 89, and 75 g/min, respectively). The corresponding case‐ and day‐level rates were 190.8 ± 87.9 g/min for ERCPs (case level, *n* = 10; range 82.1–347.4), 183.7 ± 68.8 g/min for EGDs (day level, *m* = 16; range 125.5–384.4), 106.9 ± 60.3 g/min for ESDs (case level, *n* = 10; range 28.3–205.1), and 101.1 ± 26.0 g/min for CSs (day level, *m* = 15; range 49.9–154.8). Although ESDs had the highest per‐procedure CO_2_ emissions, short‐duration EGDs and ERCPs had the highest per‐minute CO_2_ emissions. ESDs and CSs were ordered differently by both estimators: ESDs ranked above CSs by the mean case‐ and day‐level rates but below CSs by the aggregate rate, reflecting the wide variation in the ESD duration (28.7–223.1 min), which weights the aggregate rate toward the longest cases. These results are summarized in Table 2.

**TABLE 2:** Time‐adjusted waste generation and estimated incineration‐related CO_2_ emissions per gastrointestinal endoscopy. The aggregate rate was calculated as (the total waste mass or total estimated CO_2_ emissions)/(the total procedure duration in minutes) across all the measurements and is retained as the principal estimate; it is an aggregate ratio and is not the mean of the case‐ or day‐level rate. Case‐ and day‐level rates are day‐level values for esophagogastroduodenoscopies (EGDs) and colonoscopies (CSs) (the daily total divided by the total procedure duration that day across *m* measurement days) and case‐level values for endoscopic submucosal dissections (ESDs) and endoscopic retrograde cholangiopancreatographies (ERCPs) (across *n* procedures). The duration of each procedure was recorded in all four groups; for EGDs and CSs, only the waste measurements were pooled by day.

Parameter	EGD (*n* = 79, *m* = 16)	CS (*n* = 38, *m* = 15)	ESD (*n* = 10)	ERCP (*n* = 10)
**Waste mass per unit time (g/min)**				
Aggregate rate	109	75	44	95
Case‐ and day‐level rates, mean ± SD (range)	119.3 ± 40.6 (86.6–238.0)	88.7 ± 28.9 (43.3–153.5)	59.0 ± 30.7 (24.4–116.7)	105.6 ± 41.8 (58.0–183.5)
**Estimated incineration‐related CO_2_ emissions per unit time (g/min)** ^a^				
Aggregate rate	165	89	75	168
Case‐ and day‐level rates, mean ± SD (range)	183.7 ± 68.8 (125.5–384.4)	101.1 ± 26.0 (49.9–154.8)	106.9 ± 60.3 (28.3–205.1)	190.8 ± 87.9 (82.1–347.4)
**Procedure duration (min), case level**				
Mean ± SD (range)	5.8 ± 3.7 (2.2–19.5)	13.3 ± 8.4 (3.8–41.4)	77.7 ± 58.8 (28.7–223.1)	28.5 ± 9.7 (13.0–49.0)

^a^ Estimated CO_2_ emissions are only incineration‐related, derived from the chemical approximation of the combustible waste as cellulose (C_6_H_10_O_5_)*n* and the non‐combustible waste as polyethylene (CH_2_)*n*, and do not represent the full carbon footprint of each procedure. Liquid and landfill‐designated wastes are excluded from the estimation of CO_2_ emissions (see Methods and Discussion).

### 3.3 Estimated Annual Institutional CO_2_ Emissions

Applied to the annual procedure volume of Kagawa Rosai Hospital, the estimated annual GIE‐derived incineration‐related CO_2_ emissions were approximately 2200, 2070, 120, and 1410 kg for EGDs, CSs, ESDs, and ERCPs, respectively, for a total of approximately 5800 kg. Although ESDs generated the highest per‐procedure emissions, the highest annual institutional contributions were derived from EGDs and CSs, reflecting their high procedure volumes.

### 3.4 Sensitivity Analysis of Incineration‐Related CO_2_ Emissions

The per‐procedure CO_2_ emissions varied depending on the assumptions about the waste's chemical composition (Table 3). The estimated emissions were approximately 64%–69% of the base composition scenario values in the lower‐emission composition scenario (where all the incineration‐eligible waste behaved as cellulose) and 123%–134% in the higher‐emission composition scenario (where all such waste behaved as polyethylene). The per‐minute CO_2_ emissions varied within a similar relative range (49–115 and 94–221 g/min for the lower‐ and higher‐emission composition scenarios, respectively). The relative ranking of the procedures was maintained across scenarios; in each, ESDs had the highest per‐procedure emissions, and ERCPs and EGDs had the highest per‐minute emissions.

**TABLE 3:** Sensitivity analysis of per‐procedure incineration‐related CO_2_ emissions for three waste composition scenarios.

Procedure	Lower‐emission composition (all cellulose)	Base composition (current model)	Higher‐emission composition (all polyethylene)	Range (% of base)
**Per‐procedure CO_2_ emissions (g)**				
EGD	658	949	1269	69%–134%
CS	752	1178	1450	64%–123%
ESD	3807	5834	7342	65%–126%
ERCP	3264	4780	6295	68%–132%
**Per‐minute CO_2_ emissions (g/min)**				
EGD	114	165	221	69%–134%
CS	56	89	109	64%–123%
ESD	49	75	94	65%–126%
ERCP	115	168	221	68%–132%

Base composition scenario: Combustible and non‐combustible wastes are assumed to be cellulose and polyethylene, respectively.

Lower‐emission composition scenario: All the incineration‐eligible waste is assumed to be cellulose (1.63 g CO_2_/g).

Higher‐emission composition scenario: All the incineration‐eligible waste is assumed to be polyethylene (3.14 g CO_2_/g).

These scenarios only assess the uncertainty in the waste's composition and do not represent the full lower and upper bounds of the uncertainty in the emission estimates.

EGDs (*n* = 79, *m* = 16) and CSs (*n* = 38, *m* = 15) are based on daily aggregated data; ESDs (*n* = 10) and ERCPs (*n* = 10) are based on case‐level data.

*n*: Total number of procedures; *m*: number of measurement days.

## 4 Discussion

These results are the first to quantify medical waste and the associated incineration‐related CO_2_ emissions for four gastrointestinal endoscopic procedures performed in clinical settings in Japan. ESDs had the highest per‐procedure CO_2_ emissions, and both ERCPs and EGDs had the highest per‐minute emissions. Our results are particularly relevant to settings where procedures are performed frequently or depend heavily on medical devices.

The proportionally large amount of ESD‐derived landfill waste may reflect the complexity and invasiveness of ESDs. GIEs generate considerable waste, mainly because of their reliance on single‐use devices [4] and because therapeutic procedures are usually more resource‐intensive than diagnostic ones [9]; our finding that ESDs generated the most per‐procedure waste is consistent with these observations. Welsch et al. [9] reported an average of approximately 1.1 kg of waste per procedure and classified per‐procedure waste as low (<0.5 kg), moderate (0.5–1.5 kg), or high (>1.5 kg), with ERCPs in the high category. To the best of our knowledge, the ESD‐generated waste volume in Japan has not been previously evaluated; we report herein that ESDs generate more waste than ERCPs, also placing ESDs in the high category.

The high per‐minute CO_2_ emissions in ERCPs and EGDs reflect different underlying mechanisms. Because the amount of materials used per EGD is relatively constant and the procedure is fast, the time‐adjusted waste generation rate is high. However, although the procedure time for ERCPs is longer than that of EGDs, the per‐procedure waste volume of ERCPs is higher, producing a similarly high per‐minute waste generation rate. Most previous studies have evaluated the environmental impacts of GIEs per procedure without adjusting for duration, leaving differences in time‐related efficiency unexplored. Our finding that diagnostic EGDs and therapeutic ERCPs generated the highest per‐minute CO_2_ emissions is new and suggests that even short diagnostic procedures may have considerable time‐adjusted environmental impacts when the per‐case material usage is relatively fixed. Lacroute et al. [10] reported that a single examination emitted 28.4 kg of CO_2_e (carbon dioxide equivalents). Reported waste quantities vary considerably between settings [11], reaching 3.03 kg per procedure in a prospective audit of a single endoscopy unit [12]. The estimated environmental impact mainly depends on assumptions about system boundaries, waste disposal scenarios, and emission factors [13]. We focus on incineration‐associated CO_2_ emissions, estimated from measured waste volumes, because at both institutions, medical waste, other than landfill‐designated items, is incinerated.

The volume of per‐endoscopy medical waste varies by country and facility: Menini et al. [14] reported approximately 1.91 kg of regulated waste per procedure in a single Italian center; Rughwani et al. [15] reported 0.504 kg per procedure at a tertiary care facility in India, and Jung et al. [7] reported an average per‐procedure weight of 1.34 kg across multiple hospitals in South Korea (approximately 0.24 and 0.43 kg for an EGD and a CS, respectively). Our per‐procedure waste masses for an EGD and a CS exceed those of Jung et al. [7], possibly because of differences in clinical practice and in the range of disposable items included in the measurement. A direct comparison of CO_2_ values is also hampered because the South Korean study reported waste masses only, whereas our estimates are derived from incineration modeling. Our time‐adjusted emissions further suggest that both rapid diagnostic procedures with a relatively fixed material usage and longer therapeutic procedures with a high material usage may represent important targets for waste reduction interventions.

This study has several important limitations. First, we estimate CO_2_ emissions solely from the incineration of the measured waste, using simplified chemical approximations for combustible (cellulose) and non‐combustible (polyethylene) materials, and this does not reflect the overall carbon footprint of each procedure. Because emissions from upstream processes (e.g., the manufacture and transportation of disposable equipment), equipment reprocessing, water and energy consumption, and transport to disposal facilities are not included, the total environmental impact may be underestimated. Second, our sensitivity analysis (Table 3) only addresses the uncertainty in the waste's composition. Other sources of uncertainty, including alternative disposal routes, transport distances, moisture content, combustion efficiency, energy recovery, and emission factors, were not evaluated, and the scenario range should, therefore, not be interpreted as the overall lower and upper bounds of the emission estimates. Third, the numbers of ERCPs and ESDs were limited to 10 each. Because both procedures vary depending on indications, disease characteristics, lesion locations, and the equipment used, our sample size could not adequately capture their variability, limiting the generalizability of our findings. Fourth, because the waste for EGDs and CSs was pooled by day, their reported distributions describe the variability across the 16 and 15 measurement days, respectively, rather than across individual cases; the procedure duration, by contrast, was recorded for every procedure. Fifth, the contributions of the two institutions were markedly unbalanced: 131 of the 137 audited procedures were performed at Kagawa Rosai Hospital, and only six ESDs came from Kagawa University Hospital. The per‐procedure estimates for EGDs, CSs, and ERCPs are, therefore, single‐center values, and multicenter measurements with a more balanced contribution from each institution are needed before these estimates can be generalized.

Given Japan's commitment to carbon neutrality by 2050, any strategy that expands reusable equipment use, optimizes disposable item use, and develops low‐impact alternatives will contribute to this goal. Although our preliminary baseline data can support greener endoscopy and reduce the environmental impact of this sector, future studies applying full life‐cycle assessment frameworks are needed.

## 5 Use of Generative Artificial Intelligence

In preparing this manuscript, the authors used Claude (Anthropic, San Francisco, CA, USA), a generative artificial intelligence tool based on a large language model, to assist with methodological considerations, the verification of calculations, the creation of tables, and a comparison of the literature. All the conceptual content, data interpretation, and the final manuscript text were reviewed, verified, and finalized by the authors, who assume full responsibility for the integrity and accuracy of this paper. This disclosure is made in accordance with the International Committee of Medical Journal Editors’ (ICMJE's) recommendations.

## Author Contributions

**Ryosuke Kawanishi**: methodology, investigation, and writing – original draft; **Atsushi Imagawa**: data curation and formal analysis; **Naoya Tada**: data curation; **Noriko Nishiyama**: data curation; **Haruo Oba**: data curation; **Hideki Kobara**: project administration.

## Funding

No financial relationship exists with any company or organization that might have an interest in the submitted work. All the authors have completed the ICMJE disclosure form.

## Ethics Statement

This study was approved by the Clinical Ethics Committee of Kagawa University Hospital (Registration No. 2025‐074) and was conducted in accordance with the Declaration of Helsinki. The component conducted at Kagawa Rosai Hospital was referred to that institution, which determined that the study did not fall within the scope of the Japanese Ethical Guidelines for Medical and Biological Research Involving Human Subjects and that a formal ethics review, therefore, was not required there. This determination reflects the fact that the study only recorded the mass of the medical waste generated by each procedure and the duration of each procedure; no patient‐identifiable information, clinical data, or biological specimens were collected, and no intervention was performed. As this study did not involve any intervention or the use of identifiable patient data, the requirement for individual informed consent was waived by the ethics committee, and an opt‐out approach was used in accordance with institutional policy. This study was not prospectively registered in a clinical trial registry because it is an observational study of medical waste rather than a clinical trial. Furthermore, this study did not involve animals and, therefore, did not require approval related to animal experimentation.

## Conflicts of Interest

The authors declare no conflicts of interest.

## Data Availability

The data that support the findings of this study are available from the corresponding author upon reasonable request.
